# Protective role of Wallerian degeneration slow (*Wld^s^*) gene against retinal ganglion cell body damage in a Wallerian degeneration model

**DOI:** 10.3892/etm.2012.844

**Published:** 2012-11-30

**Authors:** CHENG-HU WANG, BO WANG, RI-LE WENDU, HUI-E BI, GUO-FAN CAO, CHAO JI, QIN JIANG, JIN YAO

**Affiliations:** 1 The Affiliated Eye Hospital of Nanjing Medical University; Nanjing, Jiangsu, P.R. China; 2 Department of Dermatology, The First Affiliated Hospital, Nanjing Medical University, Nanjing, Jiangsu, P.R. China

**Keywords:** Wallerian degeneration slow, axon degeneration, retinal ganglion cell body degeneration

## Abstract

Nerve distal axon injury-induced Wallerian degeneration is significantly delayed in Wallerian degeneration slow (*Wld^s^*) mutant mice, although the associated mechanisms are not completely clear and the role of *Wld^s^* in retinal ganglion cell (RGC) body damage is not fully understood. In the present study, a Wallerian degeneration model was established in wild*-*type (WT) and *Wld^s^* mutant mice by creating mechanical injury in the optic nerves. Wallerian degeneration and RGC body collapse were observed to be significantly delayed in the *Wld^s^* mice. Electroretinograms (ERG) and visual evoked potentials (VEPs) in *Wld^s^* mice were also significantly improved at the earlier stages (one week) following injury. The retina immunohistochemistry results showed that *Wld^s^* mice had more ordered cells and improved inner granular cell layer arrangement compared with the WT mice. Optic nerve Luxol Fast Blue (LFB) staining showed greater axon demyelination in WT mice than in *Wld^s^* mice. A large number of apoptotic cells were also observed in the WT mice. The present results suggest that the *Wld^s^* gene may also protect the RGC body following nerve injury.

## Introduction

Glaucoma optic nerve (ON) damage, optic neuritis and other ophthalmic neurodegenerative diseases are the leading causes of legally defined blindness in adults. The number of patients with ophthalmic neurodegenerative diseases increases annually (1). Axonal degeneration is an active neurodegeneration process, which eventually leads to permanent vision loss. Wallerian axon degeneration, as observed in numerous neurodegenerative diseases, is a commonly used model for studying axonal degeneration (2*–*4). It is caused by various fracture traumas. Broken-end distal axons are only able to live for a few days due to somatic nutritional support. The axon then quickly denatures and disintegrates. The debris is engulfed by Schwann cells and macrophages (4). Wallerian degeneration may be observed in the central nervous system (CNS) and peripheral nervous system. How it is initiated is not fully understood and the underlying molecular mechanisms remain largely unknown (2,3).

The degeneration of transected axons is significantly delayed in the Wallerian degeneration slow (*Wld^s^*) mouse strain. The phenotype is mainly due to the overexpression of a chimeric protein, Wld^s^(4). Wld^s^ is composed of a short fragment of the E4*-*ubiquitin ligase Ube4b (yeast Ufd2) and a full*-*length nicotinamide mononucleotide adenylyl*-*transferase*-*1 (Nmnat*-*1) (5,6). The underlying molecular mechanisms of the protective effects of the *Wld^s^* gene or its product on the axon are not clear, although hypotheses involving overexpression of dominant negative ubiquitin proteins and Nmnat1*-*induced biosynthesis of nicotinamide adenine dinucleotide (NAD) have been proposed (7).

At present, studies on *Wld^s^* mice have been focused on the molecular mechanisms of its effect on the delayed peripheral nerve axonal degeneration. However, few studies have focused on the effects of *Wld^s^* on retinal ganglion cell (RGC) body degeneration. In the present study, the *Wld^s^* gene was observed to not only delay the degeneration of axons, but also protect RGC bodies in a Wallerian axon degeneration model.

## Materials and methods

### Mouse ON surgery

The mouse ON crush injury was performed unilaterally under deep anaesthesia with intraperitoneal ketamine (10% urethane, 5% chloral hydrate; 30mg/kg). With the aid of an operating microscope, the superior conjunctiva was incised with spring scissors and, following blunt dissection, the carefully exposed ON was crushed for 20 sec with calibrated forceps (type 3C; Dumont, Montignez, Switzerland), 2–3 mm behind the globe. The eye on which surgery was performed was covered with ofloxacin ointment (external application). All the animal experiments undertaken in the present study were approved by the Nanjing Medical University Animal Committee.

### Electroretinograms (ERGs)

As previously reported (8), ERGs were used to monitor the overall retinal function prior to and at various time points following surgery. The wild-type (WT) and *Wld^s^* mice were prepared under dim red illumination. Animals were injected intraperitoneally with a compound anesthetic (dihydroetorphine hydrochloride, haloperidol; 0.5 ml/kg) and the pupils were dilated with 0.1% tropicamide. During the recording session, the mice were placed on a heating plate to maintain body temperature at 37°C. ERGs were recorded with an Ag-AgCl electrode placed in contact with the cornea limbus; a reference electrode was inserted in the cheek and a ground electrode was placed in the tail. The cornea was kept moist with carboxymethyl cellulose sodium. The reference and ground electrodes were stainless steel needles inserted under the skin of the scalp and tail, respectively. A small drop of balanced salt solution (Alcon, Fort Worth, TX, USA) was topically applied to the cornea to prevent dehydration for the duration of the recording. A visual stimulus of contrast-reversing horizontal bars (field area, 50x58°; mean luminance, 50 cd/m^2^; spatial frequency, 0.05 cyc/deg; contrast, 98%; temporal frequency, 1 Hz) was aligned with a projection of the pupil at the viewing distance of 15 cm. The eyes were not refracted for the viewing distance given that the mouse eye has a large depth of focus due to the pinhole pupil. Retinal signals were amplified (10,000*-*fold) and the band-pass filtered (1–30 Hz). Three consecutive responses to each of 600 contrast reversals were recorded. The responses were superimposed to check for consistency and then averaged (1,800 sweeps). The peak ERG (PERG) is a light*-*adapted response. PERGs consisting of a major positive wave followed by a slower negative wave were automatically analyzed to evaluate the response amplitude which was defined as the sum of the absolute values of the maximum and minimum voltages (peak*-*to*-*trough amplitude).

### Visual evoked potentials (VEPs)

The mice were anesthetized with ketamine (75 mg/kg) and xylazine (10 mg/kg). A normal body temperature was maintained using a surrounding hot water bag and the corneas were kept moist with saline or 2.5% hydroxypropyl methylcellulose (Johnson & Johnson, Shanghai, China). Stainless steel needle electrodes, 7 mm in length, were placed subdermally in the occipital midline (active) and the pinna. A subdermal needle electrode in the midline near the tail served as the ground electrode. The signals were amplified by 10,000-fold, filtered from 1–1000 Hz and were digitized and recorded by a Nicolet 4094 digital oscilloscope (Nicolet Instruments Technology Inc., Madison, WI, USA) set for averaging (0.396 sec trace duration, digitization rate = 10,000/sec). No artifact rejection was used. During the recording of the VEPs, the ambient illuminance was dim (2.9 lx) as measured at the eye. The surrounding luminance was 0.3*–*0.6 lx. The mice were placed prone, facing a Grass PS22 flash lamp diffusing faceplate (Grass Instruments Co., Quincy, MA, USA) at a distance of 20 cm. The mice were stimulated binocularly at 1 flash/sec. The flash illuminance at the eyes was 88 *μ*sec and flash duration was 10 *μ*sec. A total of 120 traces synchronized with flash onset were averaged to produce one VEP. Four VEPs were recorded from each mouse. Noise controls were produced in the same manner but with the flash occluded by a black opaque cloth. The digitized waveforms were saved as ASCII files with Vu*-*Point II (Maxwell Laboratories Inc., La Jolla, CA, USA).

### Retinal histopathology evaluation after ON surgery

As previously reported (8), at the completion of the ERGs, the mice were sacrificed by cervical dislocation. The two eyes were enucleated and fixed in Feteke's solution for 2 h, followed by a 12-h fixation in 4% paraformaldehyde. After dehydration in a graded ethanol series, the eyes were embedded in paraffin. Sections (5 *μ*m in thickness) were cut along the vertical meridian at 0.05 mm intervals, yielding 18 sections from each eye, and stained with hematoxylin and eosin (HE) to evaluate the retinal histology. The presence of inflammatory cell infiltration was assessed with a 4*-*point scale. No infiltration, 0; mild cellular infiltration of the ON or ON sheath, 1; moderate infiltration, 2; severe infiltration, 3; massive infiltration, 4.

### Luxol Fast Blue (LFB) staining

Sections were cut from the paraffin*-*embedded tissue. The slides were placed in LFB solution overnight at 55°C, differentiated in alcohol, dipped in 0.05% lithium carbonate solution and then counterstained with cresyl violet.

### Terminal deoxynucleotidyl transferase (TdT)-mediated dUTP nick end labeling (TUNEL) staining

A TUNEL assay was used to evaluate the apoptosis of retinal cells following ON injury. The eyes were enucleated 1, 2 and 3 weeks after surgery and the retinas were dissected as described previously. The tissue was then fixed with 10% formaldehyde for 24 h. The whole retina was divided into two parts through the optic disc and then dehydrated and embedded in paraffin. Sagittal sections (5 mm in thickness) were cut through the optic disc and mounted. The deparaffinized sections were treated with the *In Situ* Cell Death Detection kit (Roche Molecular Biochemicals, Indianapolis, IN, USA), which is based on the binding of digoxigenin*-*dUTP to the 3′*-*OH end of DNA by TdT followed by incubation with an anti*-*digoxigenin antibody conjugated to peroxidase. The sections were examined under x40 magnification. Six microscopic fields of each eye with three adjacent areas on each side of the ON head (1 mm from the ON head) were used to count the TUNEL*-*positive cells in the ganglion cell layer (GCL). The average number of TUNEL*-*positive cells in these layers per field was used for analysis.

### Electron microscopy

Nerve segments 3 and 15*–*20 mm distal to the ON transection lesion were immersion fixed with 2.5% glutaraldehyde and 2% paraformaldehyde in 0.1 M cacodylate buffer for 5*–*14 days at 4°C. Subsequently the segments were extensively washed in 0.1 M cacodylate buffer, post*-*fixed in 4% aqueous OsO_4_ and 1% uranyl acetate and then dehydrated in graded ethanol and propylene oxide. Final resin embedding was performed using Durcupan (Fluka Chemie, Buchs, Switzerland). After polymerization for 48 h at 60°C, 50*–*100 nm transverse sections were cut on a Leica ultramicrotome, mounted on formvar-coated copper grids, counterstained with uranyl acetate and lead citrate and examined with a Zeiss EM 902 transmission electron microscope.

### Statistical analysis

All data are expressed as mean ± SEM. When comparing data from the WT with *Wld^s^* mice, P<0.05 was considered to indicate statistically significant differences using one-way analysis of variance (ANOVA) with time or genotype as the independent factor. When ANOVA showed significant differences, pairwise comparisons between the means were tested by Bonferroni post*-*hoc testing (GraphPad Prism 4.0).

## Results

### Electrophysiology

**Figure 1.**
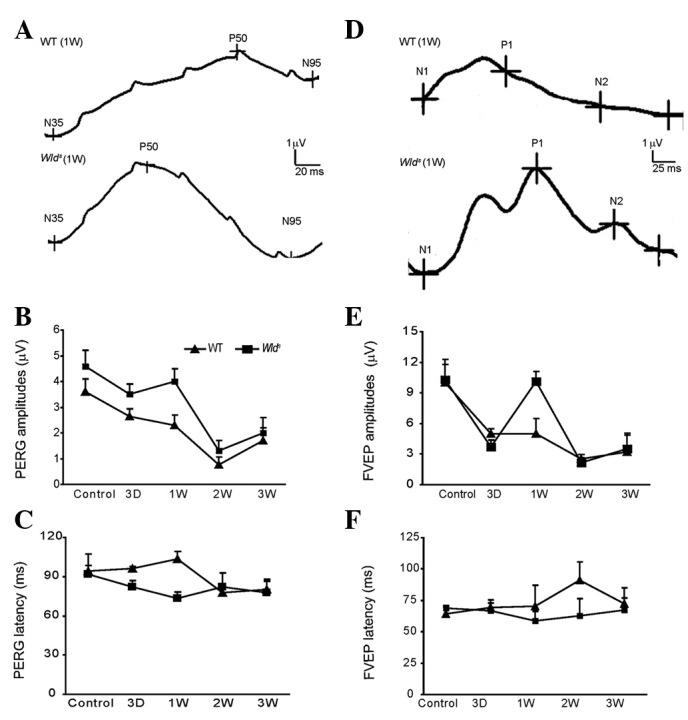
(A and D) Representative ERG waveforms of the WT and *Wld^s^* mouse before and 1, 2 and 3 weeks after ON surgery. (B, C, E and F) Amplitudes and latency of PERG before and 1, 2 and 3 weeks after ON surgery. ERG, electroretinogram; WT, wild-type; *Wld^s^*, Wallerian degeneration slow; ON, optic nerve; PERG, peak electroretinogram.

The electrophysiological properties of retina RGCs were examined first (Fig. 1A). No significant differences in the eye PERG amplitude and mean PERG latency were observed between the WT and *Wld^s^* mice prior to ON surgery. However, one week after ON surgery, the eye PERG was significantly higher in the *Wld^s^* mice (P<0.05 vs. WT) and PERG latency was also higher (P<0.05 vs. WT; Fig. 1A and B). The mean eye PERG latency was 70±3 msec in WT and 100±3 msec in *Wld^s^* mice (Fig. 1C; P<0.05). However, two weeks after ON surgery, no significant differences were observed between the WT and *Wld^s^* mice (Fig. 1A and C; P>0.05).

### VEP

Reprehensive VEPs for the WT and *Wld^s^* mice before and after ON surgery are shown in Fig. 1D. The amplitude was 10±0.3 *μ*V for the WT and *Wld^s^* mice prior to ON surgery (Fig. 1E). However, a significant difference in VEPs was observed one week after ON surgery. The mean VEPs amplitude was 5.0±0.5*μ*V for the WT mice and 10±0.5 *μ*V for the *Wld^s^* mice (Fig. 1E; P<0.05). Significant PERG latency differences were also observed two weeks after ON surgery (P<0.05; Fig. 1F). These results indicate that the *Wld^s^* gene protects the ON from axonal degeneration in *Wld^s^* mice.

### HE and TUNEL

**Figure 2.**
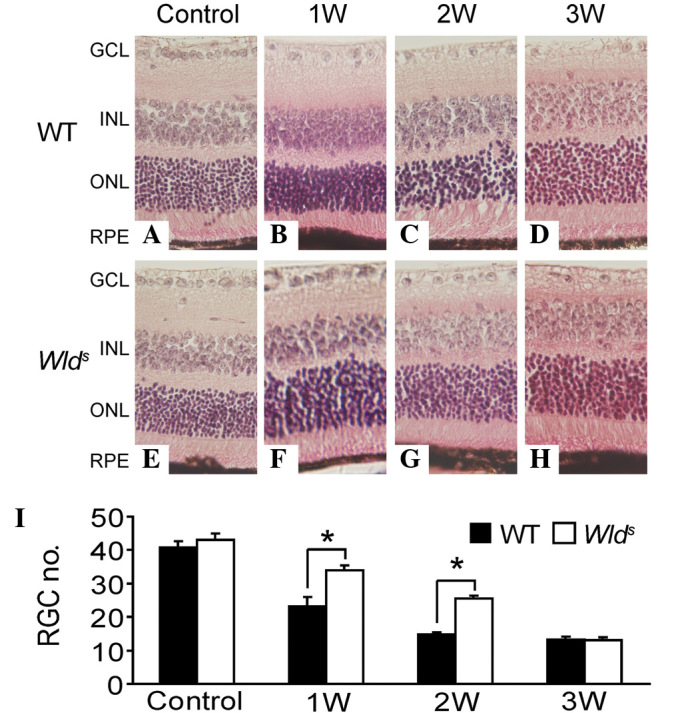
(A–H) Representative retinal HE staining of the WT and *Wld^s^* mice before and 1, 2 and 3 weeks after ON surgery (1:200). (I) Quantitative results of number of RGCs in WT and *Wld^s^* retina before or 1, 2 and 3 weeks after ON surgery. GCL, ganglion cell layer; INL, inner nuclear layer; ONL, outer nuclear layer; RPE, retinal pigment epithelium; HE, hematoxylin and eosin; WT, wild-type; *Wld^s^*, Wallerian degeneration slow; ON, optic nerve; RGC, retinal ganglion cell.

**Figure 3.**
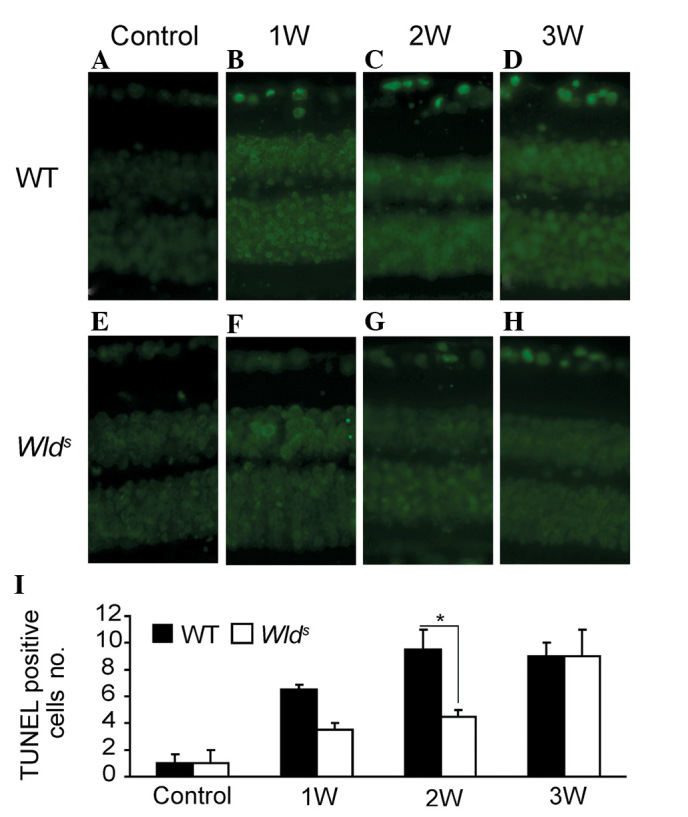
(A*–*H) Representative retinal TUNEL staining of the WT and *Wld^s^* mice before and 1, 2 and 3 weeks after ON surgery (1:200). (I) Quantitative results of relative number of TUNEL-positive cells in WT and *Wld^s^* retina before and 1, 2 and 3 weeks after ON surgery. TUNEL, terminal deoxynucleotidyl transferase (TdT)-mediated dUTP nick end labeling; WT, wild-type; *Wld^s^*, Wallerian degeneration slow; ON, optic nerve.

Prior to ON surgery, no significant difference was observed the number of RGCs between the WT and *Wld^s^* mice (Fig. 2I). One week after ON surgery, a local collapse of RGC cells was observed in the WT mice, as the number of RGCs declined significantly (Fig. 2I). By contrast, the number of RGC cells in the *Wld^s^* mice was close to that prior to surgery (Fig. 2F). Morphological observations further confirmed the results (Fig. 2A–H). Three weeks after ON surgery, the retina exhibited a marked loss of RGC in both the WT and *Wld^s^* mice (Fig. 2), with >65% of retinal pigment epithelial (RPE) cells lost in the two mouse lines (Fig. 2I). The RGC loss following the surgery appeared to result from apoptotic cell death, as increases in the number of TUNEL-positive RGC cells (a marker of cell apoptosis) were detected in the WT (at any stage) and *Wld^s^* mice (late stages; Fig. 3). Sections of whole eyes from the WT or *Wld^s^* mice were removed one, two and three weeks after surgery. TUNEL labeling was performed to stain cells undergoing active apoptosis. Few TUNEL-positive cells were observed within the RGC cell layer after one week in the *Wld^s^* mice (Fig. 3A–D), whereas a significant number of TUNEL-positive cells were present in the GCL of the WT mice (Fig. 3E–H). Three weeks after surgery, the two mouse lines showed a significant number of TUNEL-positive cells.

### ON degeneration was delayed in Wld^s^ mice

**Figure 4.**
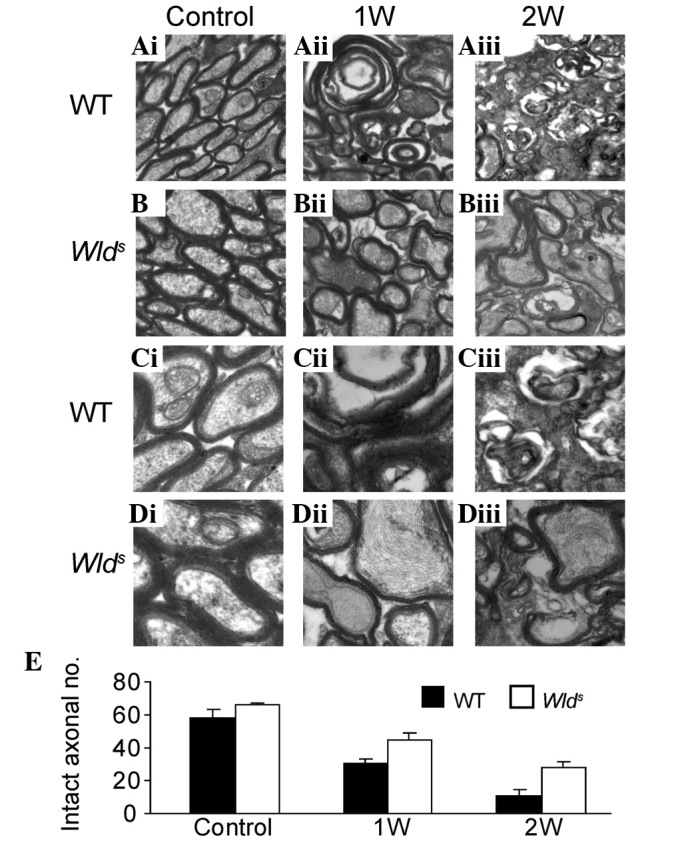
Electron micrographs from (A and C) WT and (B and D) *Wld^s^* mice before or 1 and 2 weeks after surgery. Magnifications: upper panel (A and B) 1:20,000, lower panel (C and D) 1:50,000. (E) Quantitative results of the number of intact axons before and after ON surgery in WT and *Wld^s^* mice. WT, wild-type; *Wld^s^*, Wallerian degeneration slow; ON, optic nerve.

One week after ON injury, ∼50% (for WT mice) and 70% (for *Wld^s^* mice) of myelinated axons were observed to be structurally preserved at the lesion site (Fig. 4Aii and Bii; P<0.05). Two weeks after the ON lesion was created, more intact axons were preserved in the *Wld^s^* mice than the WT mice (P<0.05; Fig. 4Aiii and Biii), with 19.0% of myelinated axons preserved in the WT mice (Fig. 3Aiii), compared with 42.0% in the *Wld^s^* mice (Fig. 4Biii; P<0.05). The ON degeneration was mostly completed within one week in the WT mice (Fig. 4Cii) and two weeks in *Wld^s^* mice (Fig. 4Diii). These results clearly demonstrate that the progression of axon degeneration was significantly delayed in injured *Wld^s^* ONs. Plastic sections prepared from degenerating WT and *Wld^s^* mouse nerves at various time points were also examined and the numbers of morphologically preserved axons were counted (Fig. 4E). Completely degenerated nerves were observed one week after injury in WT mice and two weeks in *Wld^s^* mice (Fig. 4E), suggesting that it takes ∼two weeks for the *Wld^s^* mouse ONs to complete morphological degeneration.

## Discussion

The phenotype of *Wld^s^* mice is the overexpression of a chimeric *Wld^s^* gene product, the fusion protein Nmnat1 (6), which is a key enzyme in the synthesis of NAD. Overexpression of Nmnat1 may delay the mechanical or chemical axonal injury (9) and may even have protective effects against toxic neuronal injury induced by vincristine (6). The protective effect of the gene has been demonstrated to have several aspects, including the high expression of the transgenic mouse motor nerve conduction fusion protein, synaptic transmission, vesicle cycle and lesion morphology of the motor nerve. Inhibition of the protein ubiquitination system activity may also slow Wallerian degeneration, possibly through the early stability of the axonal microtubule skeleton structure of Wallerian degeneration (10). However, the potential effects of the *Wld^s^* gene against axonal injury*-*induced RGC cell body damage are not fully understood.

Compared with the WT mice, the present results showed that the VEP amplitude was decreased in *Wld^s^* mice and the peak was delayed by at least one week following the ON damage. These results together with the the morphological changes suggest that the *Wld^s^* gene delays RGC cell degeneration. Furthermore, the immunohistochemical and electron microscopy findings in the ON demonstrated that the ON demyelination and structural disintegration in *Wld^s^* mice occurred at least one week later than in the WT mice. Similarly, the ERG amplitude in *Wld^s^* mice decreased and the PERG was delayed. The decline in the number of RGCs also occurred in the later stages (after two weeks) in *Wld^s^* mice. The RGC functional loss was delayed by one week vs. the WT mice. These results were further supported by the morphological observations. One week after the ON injury, the retina immunohisothcemical staining results showed that the volume of normal RGCs in the *Wld^s^* mice were significantly higher than that of the WT mice and the number of TUNEL-positive cells was lower. Together these results indicate that the *Wld^s^* gene delays axonal degeneration and may also be protective for RGC cell bodies.

## Abbreviations:

VEP: visual evoked potential
CNS: central nervous system
NAD: nicotinamide adenine dinucleotide
RGC: retinal ganglion cell
ERGs: electroretinograms
*Wlds*: Wallerian degeneration slow
LFB: Luxol fast blue
TUNEL: terminal deoxynucleotidyl transferase (TdT)-mediated dUTP nick end-labeling

## References

[1] Jackson GR, Owsley C (2003). Visual dysfunction, neurodegenerative diseases, and aging. Neurol Clin.

[2] Wang JT, Medress ZA, Barres BA (2012). Axon degeneration: molecular mechanisms of a self-destruction pathway. J Cell Biol.

[3] Feng Y, Yan T, He Z, Zhai Q (2010). *Wld*(S), Nmnats and axon degeneration - progress in the past two decades. Protein Cell.

[4] Wang J, He Z (2009). NAD and axon degeneration: from the *Wlds* gene to neurochemistry. Cell Adh Migr.

[5] Fernando FS, Conforti L, Tosi S, Smith AD, Coleman MP (2002). Human homologue of a gene mutated in the slow Wallerian degeneration [C57BL/*Wld(s)*] mouse. Gene.

[6] Xue L, Fletcher GC, Tolkovsky AM (1999). Autophagy is activated by apoptotic signalling in sympathetic neurons: an alternative mechanism of death execution. Mol Cell Neurosci.

[7] Fainzilber M, Twiss JL (2006). Tracking in the *Wlds-* the hunting of the SIRT and the luring of the Draper. Neuron.

[8] Yuan S, Zhang W, Ding J, Yao J, Jiang Q, Hu G (2009). Increased sensitivity to retinal light damage in aquaporin-4 knockout mice. Exp Eye Res.

[9] Araki T, Sasaki Y, Milbrandt J (2004). Increased nuclear NAD biosynthesis and SIRT1 activation prevent axonal degeneration. Science.

[10] Zhai Q, Wang J, Kim A (2003). Involvement of the ubiquitin-proteasome system in the early stages of wallerian degeneration. Neuron.

